# Discordant phenotypes in twins with infantile nystagmus

**DOI:** 10.1038/s41598-021-82368-0

**Published:** 2021-02-02

**Authors:** Abdullah Aamir, Helen J. Kuht, Rebecca J. McLean, Gail D. E. Maconachie, Viral Sheth, Basu Dawar, Ravi Purohit, Nicolas Sylvius, Michael Hisaund, Alina Zubcov-Iwantscheff, Frank A. Proudlock, Irene Gottlob, Mervyn G. Thomas

**Affiliations:** 1 The University of Leicester Ulverscroft Eye Unit, Department of Neuroscience, Psychology and Behaviour, University of Leicester, RKCSB, P.O. Box 65, Leicester, LE2 7LX UK; 2 NUCLEUS Genomics, Core Biotechnology Services, University of Leicester, Leicester, LE1 9HN UK; 3 Practice for Ophthalmology, Ginnheimer Hohl 6, 60431 Frankfurt, Germany

**Keywords:** Genetics, Neuroscience

## Abstract

Infantile nystagmus (IN) may result from aetiologies including albinism and *FRMD7* mutations. IN has low prevalence, and twins with IN are rare. Whilst discordant presentation has been previously reported for IN, we present for the first time the comprehensive assessment of diagnostically discordant monozygotic twins. From a cohort of over 2000 patients, we identified twins and triplets discordant for nystagmus. Using next-generation sequencing, high-resolution infra-red pupil tracking and optical coherence tomography, we characterised differences in genotype and phenotype. Monozygotic twins (n = 1), dizygotic twins (n = 3) and triplets (n = 1) were included. The monozygotic twins had concordant *TYR* variants*.* No causative variants were identified in the triplets. Dizygotic twins had discordant variants in *TYR*, *OCA2* and *FRMD7*. One unaffected co-twin demonstrated sub-clinical nystagmus. Foveal hypoplasia (FH) was noted in four of five probands. Both co-twins of the monozygotic pair and triplets displayed FH. In three families, at least one parent had FH without nystagmus. FH alone may be insufficient to develop nystagmus. Whilst arrested optokinetic reflex pathway development is implicated in IN, discordant twins raise questions regarding where differences in development have arisen. In unaffected monozygotes therefore, genetic variants may predispose to oculomotor instability, with variable expressivity possibly responsible for the discordance observed.

## Introduction

Nystagmus, characterised by the involuntary rhythmic oscillation of the eyes has an estimated prevalence of 24 in 10,000^1^. Infantile nystagmus (IN) is defined as nystagmus manifesting within the first 6 months of life^1,2^. IN is genetically heterogeneous, often arising from mutations of genes expressed within the developing neural retina and brain^3^. Most common causes of IN include albinism and idiopathic infantile nystagmus (IIN)^1,2^. IIN arises from *FRMD7* mutations^4,5^ and results in selective loss of horizontal optokinetic response (OKR) in humans^5,6^ and mice^7^. Disrupted OKR is associated with loss of horizontal direction selectivity in retinal ganglion cells and the transition from asymmetric to symmetric inhibitory input to horizontal direction-selective ganglion cells^7^. Although the nystagmus characteristics are similar in IIN and albinism^8^, additional phenotypical characteristics including cutaneous hypopigmentation, iris transillumination, fundus hypopigmentation, foveal hypoplasia and misrouting of retinal ganglion cell axons are seen in albinism^9^.

Twins generally share an almost identical foetal environment. Monozygotic twins share almost identical DNA, whereas dizygotic twins are usually no more genetically alike than siblings^10^. In cases of discordance, the question arises whether this is due to environmental or genetic factors. Comparisons between these twins can help elucidate developmental differences, providing an explanation for discordance. Furthermore, cases such as these raise the importance of epigenetic factors and stochastic events as the mechanisms underlying differences in phenotype. In IN, it is unclear whether arrested development of neuronal circuits at different time points may represent the cause for discordance seen in twins. Previous single case reports^11,12^ have lacked genetic diagnoses and only highlighted the presence of nystagmus in twins; its relationship to other phenotypes and discordance have not been investigated.

Using next-generation sequencing (NGS), high-resolution infra-red pupil tracking and optical coherence tomography we present a series of twins with discordant nystagmus phenotypes and discuss their aetiology.

## Results

**Figure 1:**
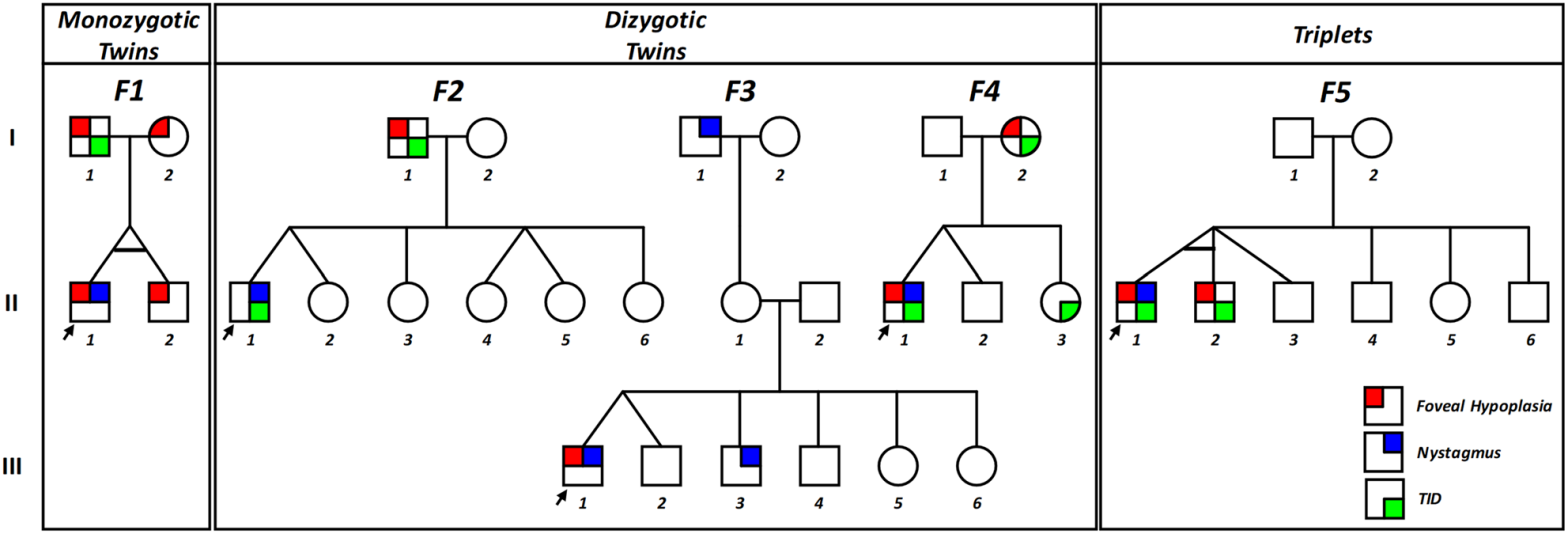
Pedigrees. Pedigrees of monozygotic twins (F1), dizygotic twins (F2, F3 and F4) and triplets (F5). The key for phenotypical characteristics shown. Arrow indicates proband. *TID* transillumination defects of the iris.

Four families with twins and one family with triplets were included in the study. Of the four twins, three were dizygotic and one monozygotic. The pedigrees of the five families are shown in Fig. 1. The summary of the clinical characteristics is shown in supplementary table 1.

### Monozygotic twins

**Figure 2:**
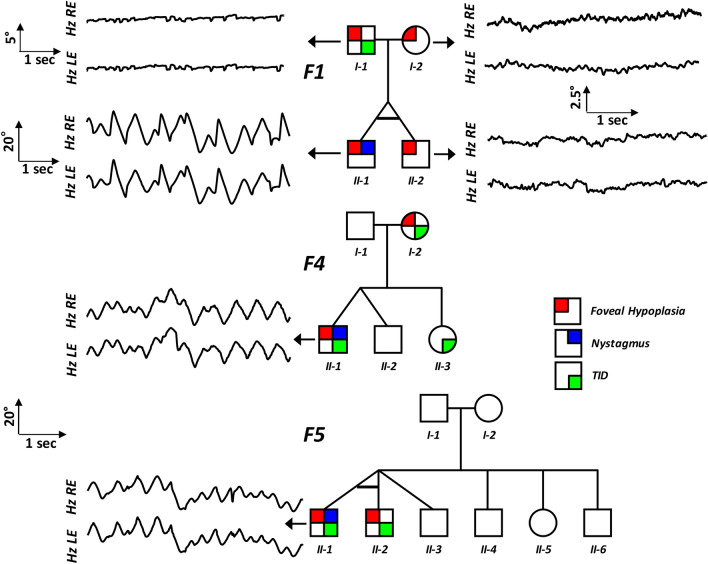
Eye movement recordings. Original eye movement recordings (EMR) in nystagmus families with monozygotic twins (F1), dizygotic twins (F2) and triplets (F5). In family F1, the proband (II-1) had high intensity nystagmus with pseudocycloid/pseudojerk waveforms. The father (I-1) had square-wave jerks and subclinical nystagmus identified on EMR. Similarly, other family members (I-2 and II-2) had subclinical nystagmus. In family F4, only the proband (II-1) had nystagmus with a pseudopendular with foveating saccades waveform. In family F5, only the proband (II-1) had nystagmus.

**Figure 3:**
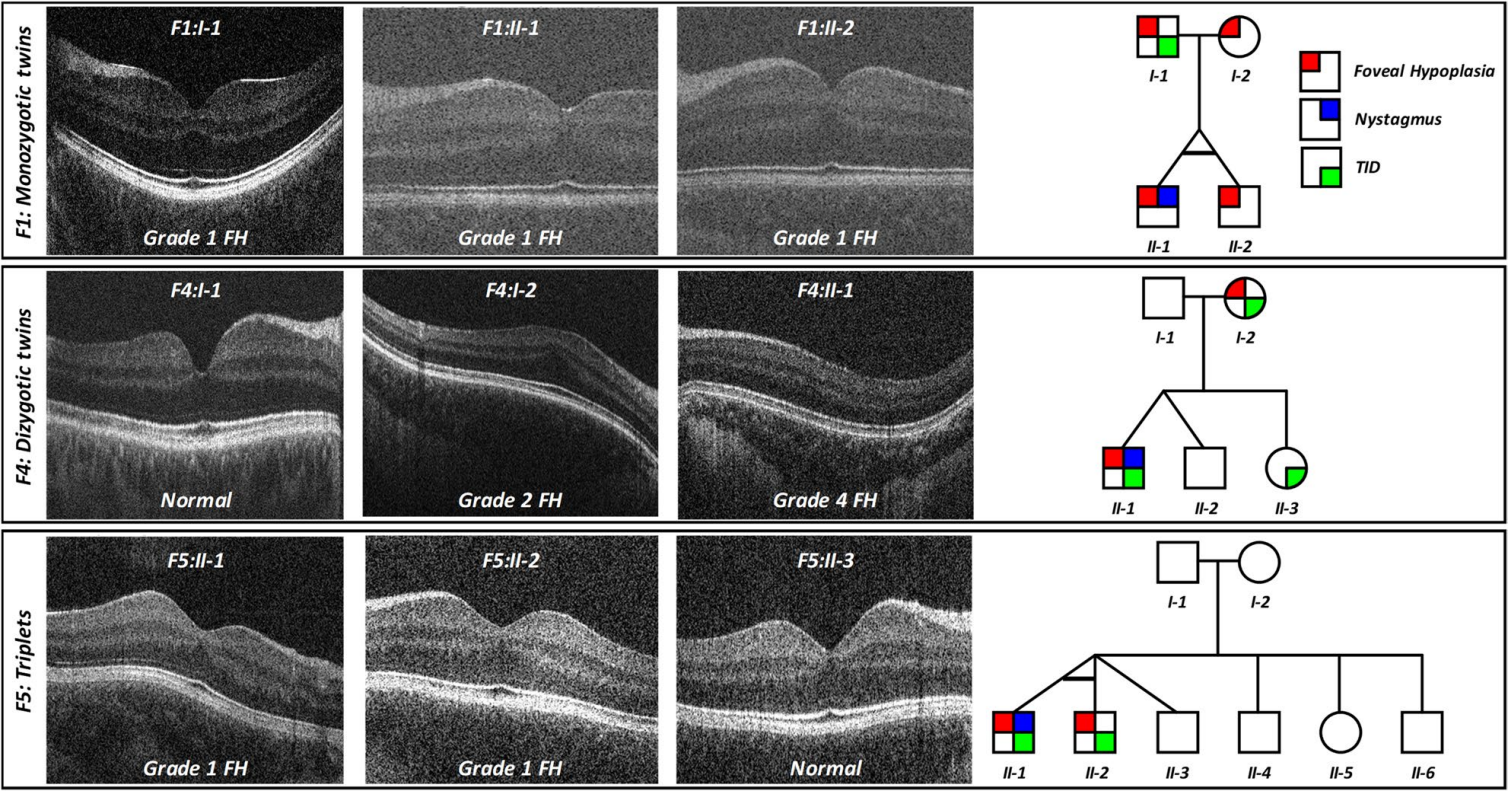
Optical coherence tomography. Spectrum of foveal development in nystagmus families. In family F1, all members had grade 1 foveal hypoplasia (FH). This is characterised by a shallow foveal pit, continuation of the inner retinal layers posterior to the foveola but a well developed outer retina. In family F4, we observe grade 2 FH in the mother (F4:I-2). This is characterised by a lack of the foveal pit but with some features of cone specialisation preserved. The proband (F4:II-1) had grade 4 FH, which has no features of foveal specialisation and resembles peripheral retina. In family F5 (triplets), the monozygotic twins in this family had grade 1 FH.

**Figure 4:**
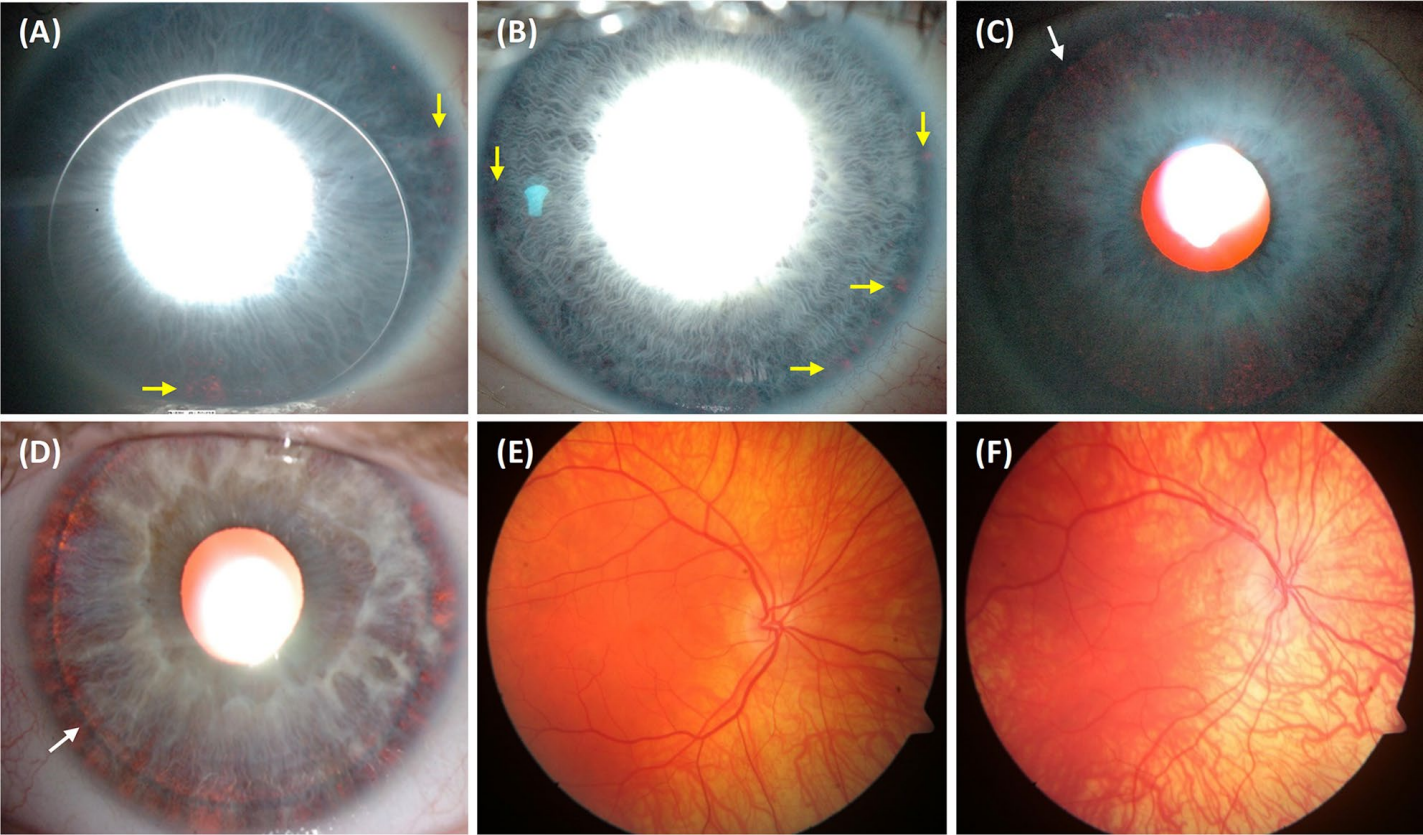
Iris and fundus photos. Spectrum of iris transillumination defects (TID) seen in the nystagmus families (**A**–**D**). Specks of TID (yellow arrows) seen in F1:I-1 (**A**) and F4:II-3 (**B**). Note the rigid gas permeable contact lens in (**A**). Higher grades of TID are seen in F4:I-2 (**C**) and F4:II-1 (**D**). There is diffuse TID with lens visible (white arrow). Varying degrees of fundus hypopigmentation in F4:I-2 (**E**) and F4:II-1 (**F**). In both fundus images there is visible choroidal vasculature predominantly in the posterior pole (**E**) and extending to the mid-periphery and towards the macula (**F**).

Twins F1:II-1 and F1:II-2 (F1, Fig. 1) were confirmed to be monozygotic by DNA zygosity analysis. The family was referred to our unit due to an unclear clinical and genetic diagnosis for F1:II-1. The twins were delivered at 33 weeks via caesarean section, with no immediate complications. Both twins had similar birthweights of 2.4 and 2.8 kg for F1:II-1 and F1:II-2, respectively. F1:II-1 was first noted to have nystagmus around age 6 months. At age 4, his nystagmus displayed characteristics compatible with IN, with increasing slow phase velocities (Fig. 2). VEP revealed misrouting in-keeping with albinism. F1:II-2 had no evidence of nystagmus. However, EMR revealed subclinical nystagmus (Fig. 2) and mild saccadic smooth pursuit. Slit lamp examination of both twins was unremarkable. Handheld OCT of both twins displayed grade 1 FH (Fig. 3). Both twins had homozygous c.1205G > A, p.(Arg402Gln) and heterozygous c.575C > A, p.(Ser192Tyr) missense *TYR* variants. The parents were also examined and underwent molecular investigations. Neither parent had significant ocular histories. The father displayed minimal transillumination defect of the iris (TID) (Fig. 4A), in-keeping with grade 1 as per the grading scheme previously described^9^. EMR highlighted subclinical frequent square wave jerks and an occasional subclinical nystagmus waveform (Fig. 2). Similarly their mother displayed fine subclinical nystagmus. Both parents had grade 1 FH on OCT (Fig. 3). Genetic analysis revealed heterozygous c.1205G > A, p.(Arg402Gln) missense *TYR* variants in both parents. Additionally, their father had a heterozygous c.575C > A, p.(Ser192Tyr) *TYR* variant*.*

### Dizygotic twins

Twins F2:II-1 and F2:II-2 were born at 37 weeks with no immediate complications. No family history of ocular disease was reported. F2:II-1, displayed conjugate horizontal nystagmus a few months after birth. EMR revealed a horizontal conjugate jerk pattern, whilst F2:II-2 displayed no nystagmus. Slit lamp examination revealed bilateral grade 1 TID in F2:II-1. OCT confirmed no FH. VEP for both twins revealed misrouting in-keeping with albinism. Both twins had reduced stereopsis, at 300″ of arc and 150″ of arc for F2:II-1 and F2:II-2 respectively. We found different heterozygous *TYR* missense variants in both twins, F2:II-1 had c.575C > A, p.(Ser192Tyr), whilst F2:II-2 had the c.1205G > A, p.(Arg402Gln) variant. Additionally, F2:II-1 had a heterozygous *OCA2* splice variant c.1503 + 5G > A. This splice variant inactivates the donor splice site and activates a cryptic donor splice site in intron 14^13^. Their father (F2:I-1) had bilateral grade 1 TID and grade 1 FH, but an otherwise unremarkable ophthalmic assessment. He had heterozygous missense variants in *TYR* (c.575C > A, p.(Ser192Tyr), c.1205G > A, p.(Arg402Gln)).

The third set of twins F3:III-1 and F3:III-2 (F3, Fig. 1) were born at 38 weeks gestation via an uncomplicated vaginal delivery. F3:III-1 was the first twin to be delivered and weighed 3.04 kg, his brother weighing 2.3 kg. Twin F3:III-1 was first noted to have horizontal conjugate jerk nystagmus in the early months following birth, however his non-identical twin brother F3:III-2 at age 4 was orthoptically unremarkable. Frisby testing revealed stereoacuity of 170″ of arc in F3:III-1. Slit lamp examination and VEPs of both eyes were unremarkable. F3:III-1 displayed grade 1 FH on OCT. No single nucleotide variants (SNV) were identified and subsequent multiplex ligation-dependent probe amplification (MLPA) analysis revealed a continuous hemizygous deletion of exons 2–12 in *FRMD7* in F3:III-1. No variants were found in his twin. The family reported nystagmus in another male sibling and the maternal grandfather (see pedigree F3, Fig. 1). This is consistent with X-linked IIN due to *FRMD7* mutation where the probands mother (F3:II-1) is an obligate carrier.

The fourth set of twins, F4:II-1 and F4:II-2 were born at 38 weeks gestation via uncomplicated vaginal delivery. F4:II-1 was initially noted to have nystagmus at 6 weeks of age, however his brother remained orthoptically unremarkable. EMR in F4:II-1 demonstrated a conjugate horizontal pseudopendular waveform with foveating saccades (Fig. 2). He lacked evidence of stereovision, with negative Frisby and Lang tests. Further examination revealed grade 3 TID and a hypopigmented fundus (Fig. 4D–F). OCT of the fovea demonstrated grade 4 hypoplasia in F4:II-1 (Fig. 3), conversely his co-twin had a normal fovea. Their sister was noted to have minimal peripheral TID in keeping with grade 1 (Fig. 4B). Their mother was found to have misrouting on VEP, grade 2 FH (Fig. 3), transillumination defects (Fig. 4C) and a hypopigmented fundus (Fig. 4E). Genetic analysis of the proband revealed heterozygous *TYR* variant (c.575C > A, p.(Ser192Tyr)) and three heterozygous *OCA2* variants: c.2055delT, p.(Phe685fs), c.2051 T > G, p.(Phe684Cys) in *cis* and c.1327G > A, p.Val443Ile in *trans*. His mother had a heterozygous *TYR* variant (c.575C > A, p.(Ser192Tyr)) and heterozygous *OCA2* variants (c.2055delT, p.(Phe685fs) and c.2051T > G, p.(Phe684Cys); in *cis*).

### Triplets

The triplet brothers were born at 32 weeks gestation via elective caesarean section. All three required neonatal intensive care unit admission for the first 4 weeks of life. None of them developed retinopathy of prematurity. There was no prior family history of ocular disease (Fig. 1). F5:II-1 and F5:II-2 were monozygotic, with one fraternal brother, F5:II-3 (F5, Fig. 1). Of the monozygotes, F5:II-1 was first noted to have nystagmus at 5 months of age, whilst his identical twin F5:II-2 did not. At age four, EMR revealed horizontal conjugate pendular nystagmus in F5:II-1 (Fig. 2), and saccadic intrusions in F5:II-2, whilst F5:II-3 was orthoptically unremarkable. F5:II-1 had reduced stereopsis at 600″ of arc, whilst both brothers demonstrated stereopsis within normal limits. Minimal TID was seen in F5:II-1 and F5:II-2. The two monozygotic brothers had grade 1 FH on OCT (Fig. 3). The father (Turkish descent) and mother (Caucasian) had unremarkable examination findings. Genetic analysis in all three did not reveal any likely pathogenic variants to explain this phenotype. The mother was found to have a heterozygous variant in *TYR* c.1205G > A, p.(Arg402Gln). The father had a heterozygous variant in *TYR* c.575C > A p.(Ser192Tyr). The triplets lacked both of these variants.

## Discussion

We describe four families with twins and one with triplets discordant for nystagmus. Considering the prevalence of IN is between 6.1 and 14 in 10,000^1,2^, encountering twins with IN is rare. However these cases present a unique opportunity to study the interplay between genetics and environment in the development of nystagmus. Our cohort of twins have been cared for together, and are likely to have shared a similar environment, therefore reducing the possibility of their post-natal environment having a profound effect on the development of the discordance observed. IN is defined as the onset of nystagmus in the first 6 months of life, during which in our cohort of twins there was no indication of large differences in their environment. Previous single case reports of twins with discordant nystagmus, have only demonstrated dissimilar characteristics of the nystagmus waveform between co-twins^11,12^. In monozygotic twins, to date, there have been no reports of one twin being affected with nystagmus while the other is unaffected. We report for the first time in monozygotic twins (F1 and F5) the presence of nystagmus in the proband while no nystagmus was present in the other twin sibling.

It is surprising to note that inspite of identical genetic variants, F1:II-1 had nystagmus but his twin sibling F1:II-2 did not. Similarly, F5:II-1 had nystagmus but his identical twin sibling F5:II-2 did not. In family F1, interestingly all family members had grade 1 FH but only F1:II-1 had nystagmus, which suggests that FH alone is insufficient to result in the development of IN. Certainly we also see that in other subjects (F2:I-1, F4:I-2 and F5:II-2) in our cohort with FH but no nystagmus. Indeed, there are previous reports of FH without nystagmus^14^. This has also been reported in female carriers of *GPR143* mutations associated with ocular albinism^15^ and in other cases of albinism^9^. Similarly, prematurity can be contributory towards developing FH. Conversely nystagmus may exist without FH, as is the case in F2:II-1. In albinism, majority of patients exhibit FH and Kruijt et al.^9^ categorise FH as a major criterion in their diagnostic criteria. Additionally, FH has been reported in cases of *FRMD7* related IIN, however to a lesser degree than that observed in albinism^16^. Whilst F2:II-1 did not display FH, the remainder of his phenotype (nystagmus, iris transillumination, misrouting on VEP and poor visual acuity) in the presence of two putative OCA variants is in keeping with a likely diagnosis of albinism.

Previous hypothetical work has alluded to the failure of sensorimotor integration^17^ and conflicting optokinetic signals^18^ for the development of nystagmus. Therefore, arrested development of the sensory system (for example, FH) can contribute to the failure of sensorimotor integration and thus lead to oculomotor instability or nystagmus. We observe subclinical eye movement abnormalities (saccadic intrusions and subclinical nystagmus) in the unaffected siblings and in some parents (Fig. 2). Similarly, in carriers of *FRMD7* mutations a subnormal optokinetic reflex (OKR) gain has been reported^6^. Unfortunately we did not assess OKR in the families, however this could provide further insight into the mechanisms of nystagmus. Previous work on the evolution of the nystagmus waveform initially reported square-wave jerks recorded at 7 weeks after birth, followed by a small pendular nystagmus (8 weeks) and then increasing slow phase velocity waveforms (10–14 weeks)^19^. Taken together, the features observed in the unaffected twin siblings may represent a more subtle form of oculomotor instability, however with no clinical consequence. This may correlate with features of early nystagmus development (such as saccadic intrusions and small pendular nystagmus), without progression to developing clinically observable nystagmus.

Dizygotic twins can have different genetic variants but share a similar prenatal environment. In F2, the proband (F2:II-1) had a heterozygous *TYR* S192Y variant and the *OCA2* splice variant, while the co-twin (F2:II-2) only had a heterozygous *TYR* R402Q variant. Interestingly the father had some phenotypical characteristics including FH and TID. Though the father had the S192Y variant, he also harboured the R402Q (in *trans*). This additional variant together with the S192Y variant is likely to have resulted in partial manifestion of the albinism phenotype. Whilst there remains some debate regarding pathogenicity of the R402Q and S192Y *TYR* variants, there is increasing evidence to suggest that, particularly in otherwise unsolved cases, these two variants are likely to contribute to the phenotype^20,21^. Gronskøv et. al. in particular report a haplotype containing both the R402Q and S192Y variants in patients with hypomorphic features^21^. The twins in F1, were noted to be light eyed and with fair skin and hair, in-keeping with a hypomorphic albinotic appearance. Indeed, functional studies have shown both variants result in reduced tyrosinase enzymatic activity by 75% and 40% for R402Q and S192Y respectively^22^. Whilst F4:II-1 was identified to be heterozygous for S192Y in *TYR*, we further identified two heterozygous mutations in *OCA2* (V443I and F684C), both of which have been reported to be pathogenic previously^23^, and support the notion of compound heterozygosity being responsible for the phenotype^24^.

We identified a large deletion in *FRMD7* in one co-twin of a set of dizygotic twins. This could explain the cause of nystagmus within the proband, and has been reported previously^3,25^. No variants or ocular findings were found in his sibling. Genetic analysis in the triplets did not identify any causative SNVs in nystagmus associated genes, thus leaving this case genetically unsolved. Previous studies have linked the development of nystagmus to aberrant development of neuronal circuits involving the OKR pathway^5,7^. In *FRMD7* mutations, the loss of asymmetrical inhibitory inputs to direction sensitive ganglion cells occurs prior to eye opening^7^. In albino ferrets the OKR is absent during all stages of development, due to loss of direction sensitive signals^26^. These studies suggest that the optokinetic defect is innate in these genetic disorders, rather than due to progressive visual pathway degeneration. Considering our discordant twin cohort, this raises the question of when development in-utero is arrested and what factors may be contributing to tip an otherwise genetically identical pair into expressing the pathological phenotype.

Twin–twin discordance has previously been presented in the literature for a range of disorders, including: behavioural, endocrine, malignant, neurological, and psychiatric^27^. Twin–twin transfusion syndrome (TTTS) is a recognised complication of multiple-order pregnancy^28^ and has been implicated in discordant neurodevelopment between twins^29^. Whilst the twins discussed here did not develop frank TTTS, microvascular shunting of blood between twins may provide one potential factor that contributed to their discordance. Epigenetic changes, such as DNA methylation and histone modifications^30^, have also been implicated as a source of discordant phenotypes in monozygotic twins. Thus, further studies are required to correlate phenotypic discordance and epigenetic status in twins with IN, particularly in those where putative genetic alterations exist.

To our knowledge this is the first such series of twins and a case of triplets discordant for IN reported to date. We find that, particularly in discordant twins, foveal hypoplasia alone is insufficient for the development of nystagmus but may contribute to failure of sensorimotor integration. Taking into account the manner in which the nystagmus waveform evolves after birth, the question remains, what factors are protecting the unaffected co-twin from developing clinically detectable nystagmus observed in their sibling. Though we do not observe the phenotype in presumed unaffected monozygotes, EMRs detect a subclinical nystagmus waveform suggesting that the genetic variants may predispose to oculomotor instability, but with variable expressivity. We only captured known nystagmus genes for this study, thus discordance may also arise from differences in variants within genes or *cis*-regulatory elements yet to be characterised. Future experiments or investigations assessing development of OKR, whole genome sequencing, expression analyses (tissue specific RNA-Seq studies), epigenetic status and somatic mosaicism may be important to distinguish underlying factors responsible for discordant nystagmus phenotypes in twins.

## Methods

In a cohort of over 2000 patients presenting with IN, examined between 2010 and 2019, we identified five families with twins or triplets with IN. All patients were identified from the paediatric and neuro-ophthalmology clinics at the University Hospitals of Leicester. The study obtained full ethical approval from the National Research Ethics Service (Leicestershire, Northamptonshire & Rutland Research Ethics Committee; REC reference: 10/H0406/74). The study adhered to the tenets of the Declaration of Helsinki and informed consent was obtained from all participating subjects.

All participants underwent a detailed ophthalmological and orthoptic assessment including slit-lamp biomicroscopy, eye movement recordings (EMR) (EyeLink II and EyeLink1000, SR Research Ltd, Ontario, Canada), full field electroretinogram (ERG) response, visual evoked potentials (VEPs), retinal optical coherence tomography (OCT) using a handheld device (Leica Microsystems, Wetzlar, Germany, < 4-μm axial resolution) and fundus photography, where available. Binocular vision was examined using the Lang test. If the Lang test was positive, the Frisby test was used to investigate the level of stereopsis. Bagolini striate glasses were used when the Lang test was negative. ERG and VEP were recorded based on International Society for Clinical Electrophysiology of Vision (ISCEV) standards. Foveal hypoplasia (FH) was graded according to a scheme previously described^31^. Saliva (OG-500, DNA Genotek Inc., Ottawa, Ontario, Canada) samples (F1, F2, F3, F4, F5) were obtained from the proband and family members (for segregation analysis). An additional blood sample was obtained from the proband in family F3 due to a poor-quality saliva sample. DNA was extracted using the Qiagen DNA extraction kits as per the manufacturer's recommendations. NGS and segregation analysis was performed using our nystagmus panel, as previously described^3^. Briefly, DNA was randomly fragmented with base pair peak of 200–250 bp, subsequently adapters were ligated to both ends of the resulting fragments. DNA was amplified by ligation-mediated PCR, purified and hybridised to NimbleGen Human custom array (NimbleGen SeqCap EZ Choice, Roche Nimblegen Inc., Madison, WI, USA) for enrichment. NimbleDesign was used to create a custom probeset design for a nystagmus panel, including 336 known genes for IN. Probes were designed to capture exons and 40 bases flanking the splice junction. Resulting libraries were sequenced using a HiSeq 2000 (Illumina, San Diego, CA, USA) according to the manufacturers recommendation for a paired-end protocol. Mean coverage depth ranged between 180 × and 301 ×. Allelic variants were reported according to the Human Genome Variation Society guidelines. Single-nucleotide variants and indels were detected using GATK. Further annotation and filtering was performed using ANNOVAR. FishingCNV v2.1 was used for copy number variant analysis. We identified rare variants by focussing on protein-altering and splice-site changes with an allele frequency of < 1% in the 1000 genomes project or in the NHLBI ESP exomes. Variants that were previously established to cause IN were included and classified as pathogenic even if allele frequency was > 1%.

## Supplementary Information

Supplementary Table S1.

## Data Availability

We have deposited the variant data in Leiden Open Variation Database (LOVD) (available at: https://www.lovd.nl/3.0/home). The relevant accession IDs are as follows: F1:II-1 (#00324429), F1:II-2 (#00324430), F2:II-1 (#00324431), F3:III-1 (#00324433), F4:II-1 (#00324434).
