# Supplementary material for: Discordant phenotypes in twins with infantile nystagmus
Source: Sci Rep. 2021 Feb 2;11:2826. doi: 10.1038/s41598-021-82368-0 (PMC7854608; doi:10.1038/s41598-021-82368-0)
Supplement: Supplementary file 1 — Supplementary Table S1. [file 41598_2021_82368_MOESM1_ESM.pdf]

Title: Discordant phenotypes in twins with infantile nystagmus

Authors:

Abdullah Aamir[1a], Helen J. Kuht[1a], Rebecca J. McLean[1], Gail D.E. Maconachie[1], Viral Sheth[1], Basu Dawar[1], Ravi Purohit[1], Nicolas Sylvius[2], Michael Hisaund[1], Alina Zubcov-Iwantscheff[3], Frank A. Proudlock[1], Irene Gottlob[1*], Mervyn G. Thomas[1*]

Affiliations:

[1]The University of Leicester Ulverscroft Eye Unit, Department of Neuroscience, Psychology and Behaviour, University of Leicester, RKCSB, PO Box 65, Leicester LE2 7LX, UK. [2]NUCLEUS Genomics, Core Biotechnology Services, University of Leicester, Leicester LE1 9HN, UK.

[3]Practice for Ophthalmology, Ginnheimer Hohl 6, 60431, Frankfurt, Germany

[a] These authors contributed equally to this work.

*Corresponding authors, Tel: +44 (0)116 252 5879, Fax: +44 (0)116 223 1996, email: mt350@le.ac.uk and ig15@le.ac.uk

Supplementary table 1: Clinical and genetic characteristics of twins with nystagmus

			Corrected VA													
ID	Gender	Age	RE	LE	Type of Strabismus	Stereopsis	Nystagmus	Eye movement characteristics	Altered Head Position	Iris Transillumination Defect[1]	Fundus Hypopigmentation	Foveal Hypoplasia[2]	ERG	VEP	Working Diagnosis	Genetic Analysis
F1:II-1	M	5	0.65	0.75	OT	Nil	Present	HPC/J	Present	N	N	Grade 1	N	Misrouting	Albinism	TYR:p.R402Q (Homozygous), TYR:p.S192Y (Heterozygous)
F1:II-2	M	5	0.10	0.10	OT	55"	Absent	SCN	Absent	N	N	Grade 1	N	Inconclusive	Albinism	TYR:p.R402Q (Homozygous), TYR:p.S192Y (Heterozygous)
F2:II-1	M	9	0.575	0.6	XT	300"	Present	HJ	Present	Grade 1	N	N	N	Misrouting	Albinism	TYR:p.S192Y (Heterozygous), OCA2 (c.1503+5G>A) (Heterozygous)
F2:II-2	F	9	0.15	0.225	OT	150"	Absent	N	Absent	N	N	N	N	Misrouting	?Albinism/Carrier	TYR:p.R402Q (Heterozygous)
F3:III-1	M	10	0.425	0.425	OT	170"	Present	HJ	Absent	N	N	Grade 1	N	N	IIN	FRMD7: Deletion of exons 2-12 (Hemizygous)
F3:III-2	M	4	0	0	NP	NP	Absent	N	Absent	NP	NP	NP	NP	NP	N	No clear variants identified
F4:II-1	M	5	0.85	0.9	OT	Nil	Present	HP	Present	Grade 4	Grade 3	Grade 4	Inconclusive	Inconclusive	Albinism	TYR:p.S192Y, OCA2:p.V443I, OCA2:p.F685fs, OCA2:p.F684C (Heterozygous)
F4:II-2	M	5	0	0	OT	NP	Absent	N	Absent	NP	NP	N	NP	NP	N	No clear variants identified
F5:II-1	M	4	0.875	0.875	OT	600"	Present	HJ/HP	Present	Grade 1	N	Grade 1	N	Inconclusive	IIN	No clear variants identified
F5:II-2	M	4	-0.01	-0.075	OT	85"	Absent	N	Absent	Grade 1	N	Grade 1	Inconclusive	Inconclusive	N	No clear variants identified
F5:II-3	M	4	-0.025	-0.025	OT	85"	Absent	N	Absent	N	N	N	NP	NP	N	No clear variants identified

VA = visual acuity in logMAR
RE = right eye
LE = left eye
ET = esotropia
XT = exotropia
OT = orthotropia
HP = horizontal pendular nystagmus
HJ = horizontal jerk nystagmus
HPC/J = horizontal pseudocycloid/jerk nystagmus
IIN = Idiopathic infantile nystagmus
SCN = subclinical nystagmus
N = normal
NP = not performed

1. Kruijt CC, de Wit GC, Bergen AA, Florijn RJ, Schalij-Delfos NE, van Genderen MM. The Phenotypic Spectrum of Albinism. Ophthalmology 2018; 125:1953–1960.
2. Thomas MG, Kumar A, Mohammad S, Proudlock FA, Engle EC, Andrews C, et al. Structural grading of foveal hypoplasia using spectral-domain optical coherence tomography a predictor of visual acuity? Ophthalmology 2011; 118:1653–1660.
